# (*S*)‐3‐aminopiperidine‐2,6‐dione is a biosynthetic intermediate of microbial blue pigment indigoidine

**DOI:** 10.1002/mlf2.12023

**Published:** 2022-06-21

**Authors:** Zhilong Zhang, Pengwei Li, Min Wang, Yan Zhang, Bian Wu, Yong Tao, Guohui Pan, Yihua Chen

**Affiliations:** ^1^ State Key Laboratory of Microbial Resources, Institute of Microbiology Chinese Academy of Sciences Beijing China; ^2^ University of Chinese Academy of Sciences Beijing China; ^3^ Tianjin Key Laboratory for Modern Drug Delivery and High‐Efficiency, School of Pharmaceutical Science and Technology Tianjin University Tianjin China

**Keywords:** (*S*)‐3‐aminopiperidine‐2,6‐dione, biocatalyst, blue pigment, indigoidine, thalidomide

## Abstract

The biosynthetic investigations of microbial natural products continuously provide powerful biocatalysts for the preparation of valuable chemicals. Practical methods for preparing (*S*)‐3‐aminopiperidine‐2,6‐dione (**2**), the pharmacophore of thalidomide (**1**) and its analog drugs, are highly desired. To develop a biocatalyst for producing (*S*)‐**2**, we dissected the domain functions of IdgS, which is responsible for the biosynthesis of indigoidine (**3**), a microbial blue pigment that consists of two **2**‐like moieties. Our data supported that the L‐glutamine tethered to the indigoidine assembly line is first offloaded and cyclized by the thioesterase domain to form (*S*)‐**2**, which is then dehydrogenated by the oxidation (Ox) domain and finally dimerized to yield **3**. Based on this, we developed an IdgS‐derived enzyme biocatalyst, IdgS‐Ox* R539A, for preparing enantiomerically pure (*S*)‐**2**. As a proof of concept, one‐pot chemoenzymatic synthesis of **1** was achieved by combining the biocatalytic and chemical approaches.

## INTRODUCTION

**Figure 1:**
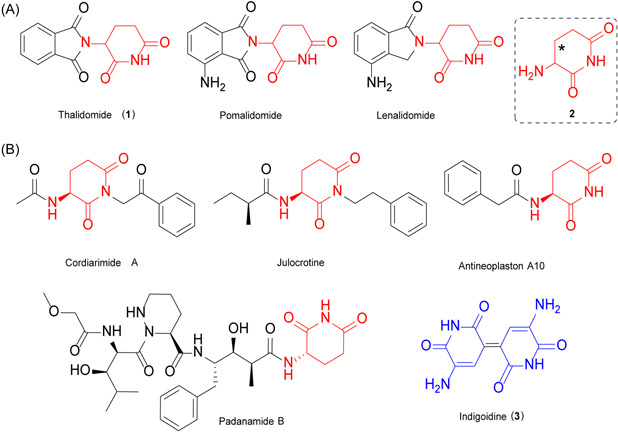
Structures of representative drugs and natural products containing **2** or **2**‐like moiety. (A) Representative drugs; (B) Natural products. **2** is marked in red and **2**‐like moiety is marked in blue.

Microbial natural products are one of the prominent sources of pharmaceuticals, agricultural antibiotics, and many other valuable small molecules. Investigation of enzymes involved in the biosynthesis of different natural products continuously provides robust biocatalysts for preparing diverse chemicals with practical use. Thalidomide (**1**; Figure 1) was approved as a drug called “Contergan” for the treatment of morning sickness during pregnancy in the 1950s and was banned in the 1960s for causing severe congenital birth defects^1^. Later studies revealed that **1** is a racemate. Its *R* enantiomer exhibits sedative and antiemetic activity, while its *S* enantiomer causes teratogenesis. Notably, the stereogenic center of **1** is present in its pharmacophore 3‐aminopiperidine‐2,6‐dione (**2**)^2^, ^3^, ^4^. After this tragedy, chiral drugs have gradually become dominant in the newly approved medicines^5^, ^6^. Ironically, owing to the immunomodulatory and antiangiogenic activities of their *S* enantiomers, **1** and its analogs, lenalidomide and pomalidomide (Figure 1A), have resurfaced as drugs for the treatment of leprosy, multiple myeloma, myelodysplastic syndrome, and mantle cell lymphoma since the 1990s^7^, ^8^, ^9^, ^10^. In practice, **1** and its analog drugs are still used as racemates to date, mainly due to the difficulties in preparing enantiomerically pure (*S*)‐**2** ^11^, ^12^, and in avoiding its racemization during drug production and administration^2^, ^13^. In addition, (*S*)‐**2** is the pharmacophore of several potential drugs targeting protein degraders (e.g., ARV‐471 and CC‐92480).

There are many synthetic routes for the preparation of **2** ^14^, ^15^, ^16^, ^17^. However, it is still a challenge to chemically synthesize (*S*)‐**2** with high enantiomeric purity^11^, ^12^. Enzymatic reactions usually exhibit high stereo‐specificity and are environmentally friendly. Therefore, developing enzymes into biocatalysts would be an attractive way to generate chiral products like (*S*)‐**2**.

Nature provides a reservoir of great chemical diversity for exploiting desired structures and reactions. Indeed, the structure of (*S*)‐**2** was found in several natural products, for example, cordiarimides^18^, julocrotine^19^, antineoplaston A10^20^, and padanamide B^21^ (Figure 1B). Unfortunately, the mechanism for the formation of their (*S*)‐**2** moieties remains elusive. In addition to these compounds, we also noticed an “old” natural product indigoidine^3^, which was first described as a microbial blue pigment as early as the 1890s, and was assigned as a dimer of two **2**‐like moieties in the 1960s (Figure 1B)^22^, ^23^. Its biosynthetic gene that encodes a single module nonribosomal peptide synthetase (NRPS) has been identified in several microorganisms, including the *idgS* gene discovered by us from *Streptomyces lavendulae* CGMCC 4.1386^24^, ^25^, ^26^, ^27^, ^28^. Taking advantage of the visible feature of **3**, its biosynthetic genes or enzymes have been developed into varied screening^27^, ^29^, ^30^, and reporter systems^31^, ^32^, biosensors for L‐glutamine (L‐Gln)^33^, and even genetic parts for breeding transgenic blue flowers^34^.

**Figure 2:**
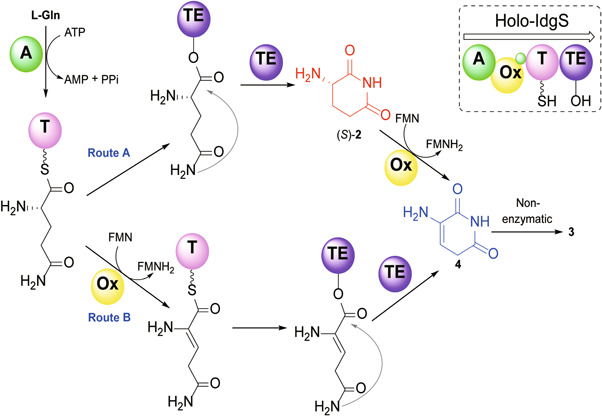
Two possible routes for the biosynthesis of **3**. Route A, L‐Gln tethered by T domain is first cyclized by the TE domain and then dehydrogenated by the Ox domain to afford **4**; route B, dehydrogenation of the tethered L‐Gln is followed by TE‐catalyzed cyclization to form **4**. Conversion of **4** to **3** was proposed to be a nonenzymatic process. L‐Gln, L‐glutamine; Ox, oxidation; TE, thioesterase.

It has been established that the single module **3** synthetase can accomplish the biosynthesis of **3** using L‐Gln as a precursor^25^. As depicted in Figure 2 using *holo*‐IdgS as an example, the adenylation (A) domain initiates **3** synthesis by loading L‐Gln to the phosphopantetheinyl arm of the thiolation (T) domain. Depending on the reaction sequence catalyzed by the oxidation (Ox) and the thioesterase (TE) domains, there are two possible routes for the IdgS‐catalyzed conversion. In route A, the tethered L‐Gln is first offloaded and cyclized by the TE domain to form **2**, which is then dehydrogenated by the FMN‐dependent Ox domain to generate **4**, whereas in route B, the tethered L‐Gln is first oxidized, and then offloaded and cyclized to give **4**. The last oxidative coupling step that dimerizes **4** to **3** was proposed to be a nonenzymatic reaction. If **3** is biosynthesized through route A, the TE domain will be a natural catalyst for generating **2**. Moreover, since the substrate is L‐Gln, the cyclized product will be the desired *S* enantiomer so long as the TE domain has no racemase or epimerase activity.

In this study, we showed that **3** can be biosynthesized via route A. An IdgS‐based biocatalyst for enantiomerically pure (*S*)‐**2** preparation was then developed and used for one‐pot chemoenzymatic synthesis of **1**.

## RESULTS AND DISCUSSION

### Detection of FMN changes in IdgS and IdgS‐TE* S1102A under oxygen‐free conditions

**Figure 3:**
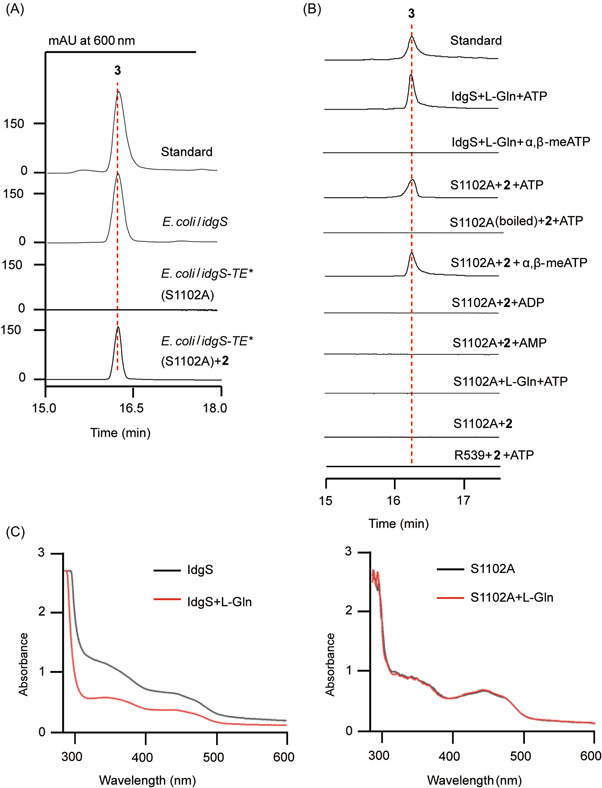
*In vivo* and *in vitro* characterization of IdgS‐TE* S1102A. (A) HPLC analysis of the metabolites of *Escherichia coli* strains with *idgS* or idgS‐TE* (S1102A). (B) HPLC analysis of the IdgS and IdgS‐TE* S1102A assays using **2** or L‐Gln as a substrate. Compound **3** was formed by IdgS using L‐Gln as a substrate. IdgS‐TE* S1102A could catalyze the conversion of **2** to **3** with the addition of ATP or *α*,*β*‐methylene adenosine 5′‐triphosphate (*α*,*β*‐meATP). (C) UV‐vis spectra of the reactions of IdgS (left) and IdgS‐TE* S1102A (right) with L‐Gln as a substrate under anaerobic conditions. HPLC, high‐performance liquid chromatography; UV, ultraviolet; vis, visible.

We first constructed a recombinant strain *Escherichia coli*/*idgS* that could produce **3** by coexpression of the *idgS* gene and *sfp* gene in *E. coli* BL21 (DE3) (Figures 3A and S1A). The phosphopantetheinyl transferase gene *sfp* was introduced for the posttranslational activation of *apo*‐IdgS to its *holo*‐form^27^, ^34^. The *N*‐His_6_‐tagged IdgS purified from *E. coli*/*idgS* could convert L‐Gln to **3** efficiently (Figures 3B and S2A). To determine whether IdgS synthesizes **3** via route A, we constructed the *E. coli*/idgS‐TE* (S1102A) strain, in which the TE domain of IdgS was inactivated by a point mutation on its substrate‐binding serine residue (S1102). Production of **3** was totally abolished in this strain, confirming the critical role of the TE domain (Figure 3A). *N*‐His_6_‐tagged IdgS‐TE* S1102A was then purified as a yellow protein as IdgS, indicating that IdgS‐TE* S1102A folded properly with FMN binding to its Ox domain (Figure S2A). In the IdgS‐catalyzed reactions, no matter through route A or B, the Ox domain will catalyze the dehydrogenation of its substrate by reducing FMN to FMNH_2_, which can be re‐oxidized to FMN by air to drive the reaction cycle. Specifically, if the reactions are performed under anaerobic conditions, the FMN will be stuck at its reduced form after one turnover, which can be monitored by the ultraviolet (UV) absorption changes of IdgS, especially at 450 nm (Figure 3C). While for IdgS‐TE* S1102A, such UV absorption changes will not take place if route A is adopted, because the catalytic cascade will be blocked before the dehydrogenation step. When we monitored the IdgS‐TE* S1102A catalyzed reactions under anaerobic conditions, no obvious change at *A*_450_ was observed, implying that no dehydrogenation occurred and IdgS‐catalyzed biosynthesis of **3** should proceed via route A.

### IdgS‐Ox domain catalyzes the oxidation of **2** to **3**

Subsequently, compound **2** was fed to *E. coli*/*idgS‐TE* * (S1102A) as an intermediate, and as expected, it was converted to **3** successfully (Figure 3A), further supporting route A. To confirm the function of the Ox domain, we prepared truncated IdgS‐Ox domain (residues 432–929) as an *N*‐His_6_‐tagged protein (Figure S2A) and tested its catalytic activity towards **2**. Unfortunately, no conversion of **2** by IdgS‐Ox was detected probably owing to impaired protein integrity. The Ox domain of **3** synthetases like IdgS is embedded in the A domain, splitting the A domain into an N‐terminal part (1–525 residues in IdgS) and a C‐terminal part (864–929 residues in IdgS). It was shown previously that the function of the domains embedded in the A domain significantly relies on the integrity of NRPS modules, which emphasizes the difficulty in investigating IdgS‐Ox as a single domain protein^35^.

Therefore, we turned to study the dehydrogenation activity of IdgS‐Ox *in vitro* using IdgS‐TE* S1102A under different assay conditions (Figures 3B and S4). Unexpectedly, IdgS‐TE* S1102A could convert **2** to **3** only with the addition of adenosine triphosphate (ATP) (Figure 3B). In addition, *apo*‐IdgS‐TE* S1102A (with no phosphopantetheine prosthetic arm at its T domain) could convert **2** to **3** when ATP was added, also supporting that IdgS‐Ox could take **2** as a substrate. In theory, ATP is only needed by the A domain of IdgS for activating and loading L‐Gln to its T domain and should not affect the activity of IdgS‐Ox domain. While in the case of IdgS, the Ox domain is embedded in the A domain, and the conformation of IdgS‐A may influence the activity of IdgS‐Ox by regulating its substrate entry and/or catalysis. Such phenomena have been observed in several different domains that are embedded in A domain^36^, ^37^, ^38^, ^39^. We envisaged that, in the IdgS‐TE* S1102A assay, ATP was used as a factor to keep the enzyme in a proper conformation rather than as a substrate of the reaction. This hypothesis was supported by the fact that (i) ATP was not consumed by IdgS‐TE* S1102A during the conversion of **2** to **3** (Figure S3); (ii) in the IdgS‐TE* S1102A assays, the productivity of **3** was not influenced by increasing ATP concentration (Figure S4A); and (iii) in the *apo*‐IdgS‐TE* S1102A assays, the productivity of **3** was not influenced by increasing ATP concentration (Figure S4B). Furthermore, to show unambiguously that ATP is a ligand instead of a cosubstrate participating in the conversion of **2** to **3**, ATP was replaced by *α*,*β*‐methylene adenosine 5′‐triphosphate (*α*,*β*‐meATP) in the IdgS‐TE* S1102A assay. The enzyme with the analog still converted **2** to **3** efficiently, strongly supporting that ATP or *α*,*β*‐meATP can induce the conformation change of IdgS‐TE* S1102A and keeps the Ox domain of the enzyme in a proper status to perform the dehydrogenation of **2** (Figure 3B).

### IdgS‐TE domain catalyzes the formation of **2**

**Figure 4:**
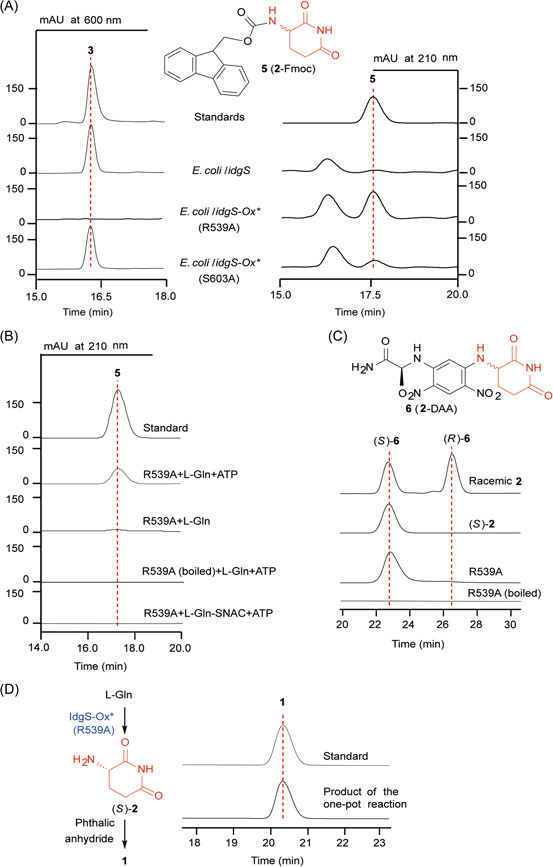
Characterization of (*S*)‐**2** as an intermediate of **3** biosynthesis and chemoenzymatic synthesis of **1**. (A) HPLC analysis of the metabolites of *E. coli*/idgS‐Ox* mutant strains (left) and the same metabolites treated with Fmoc‐Cl (right). **5** is the derivatized product of **2** with Fmoc‐Cl. (B) HPLC analysis of the IdgS‐Ox* R539A‐catalyzed reactions treated with Fmoc‐Cl. (C) HPLC analysis of the reaction mixture of IdgS‐Ox* R539A treated with FDAA. **6** is the derivatized product of **2** with FDAA. The synthetic (*S*)‐**2** was used as a standard for derivatization. (D) Chemoenzymatic synthesis of **1** using L‐Gln as a precursor. Two‐step one‐pot reaction contains IdgS‐Ox* catalyzed (*S*)‐**2** formation and subsequent chemical synthesis of **1** with phthalic anhydride (left); HPLC analysis of the one‐pot reaction (right). FDAA, 1‐fluoro‐2,4‐dinitrophenyl‐5‐L‐alanine amide; Fmoc‐Cl, 9H‐fluoren‐9‐ylmethyl carbonochloridate.

If **3** is biosynthesized via route A, the TE domain of IdgS is responsible for the cyclization of the T domain tethered L‐Gln to form **2**. At first, we tried to obtain truncated IdgS proteins with only the TE domain or T‐TE didomain, and only the latter could be expressed as a soluble protein in *E. coli* (Figure S2A). We then prepared both *apo*‐ and *holo*‐forms of the T‐TE didomain proteins and tested their activities using L‐Gln‐S‐*N*‐acetyl‐cysteamine (L‐Gln‐SNAC; Figure S5) as a substrate, but failed to detect any formation of **2**. As the truncations destroyed the integrity of IdgS and might cause improper folding of the resultant proteins, we turned to the point mutated IdgS‐Ox* and constructed two *E. coli*/idgS‐Ox* strains, (R539A) and (S603A), in which the conserved residues that were postulated to be involved in FMN binding (R539) and catalysis (S603) of the Ox domain were mutated to alanine, respectively (Figure S2B). Fermentation analysis showed that **3** production was totally abolished in *E. coli*/idgS‐Ox* (R539A), but not affected obviously in *E. coli*/idgS‐Ox* (S603A) (Figure 4A). Meanwhile, to facilitate the detection of **2**, the samples were also treated with 9H‐fluoren‐9‐ylmethyl carbonochloridate (Fmoc‐Cl) before being subjected to high‐performance liquid chromatography (HPLC) analysis with Fmoc‐derivatized **2** (**5**) as an authentic standard. To our delight, the HPLC peaks of **5** could be observed in both *E. coli*/idgS‐Ox* strains, but barely detectable in *E. coli*/*idgS* (Figures 4A and S1B). Consistent with the **3** production results, *E. coli*/idgS‐Ox* (R539A) could yield a considerable amount of **2**, while much less was accumulated in *E. coli*/idgS‐Ox* (S603A).

### Identification of the stereochemistry of **2** prepared by IdgS‐Ox* R539A

To verify the function of IdgS‐TE domain, we purified the *N*‐His_6_‐tagged IdgS‐Ox* R539A protein (Figure S2A). In contrast to the yellow IdgS, IdgS‐Ox* R539A was colorless, supporting the predicted role of R539 as an essential residue for FMN binding. The following enzymatic studies showed that IdgS‐Ox* R539A could efficiently convert L‐Gln into **2** when ATP was added for substrate activation (Figure 4B). To determine the stereochemistry of the enzymatic product, we used both racemic **2** and chemically synthesized (*S*)‐**2** as standards for comparison. These samples were treated with Marfey's reagent, 1‐fluoro‐2,4‐dinitrophenyl‐5‐L‐alanine amide (FDAA), to derivatize **2** to **6** (**2**‐DAA) before HPLC analysis (Figures 4C and S1C). As expected, the product of the IdgS‐Ox* R539A catalyzed reaction was determined to be enantiomerically pure (*S*)‐**2** (enantiomeric excess [*ee*] >99%), whose FDDA derivative had the same retention time as the authentic (*S*)‐**6** and could be well separated from (*R*)‐**6** (Figure 4C). The apparent kinetic analysis of IdgS‐Ox* R539A was also performed (Figure S6). These results not only confirmed that **3** can be biosynthesized via route A but also suggested that IdgS‐Ox* R539A can be used as a potential biocatalyst for (*S*)‐**2** preparation.

Notably, PadE‐TE, the TE domain of padanamide B biosynthetic enzyme, catalyzes a similar intramolecular cyclization of glutamine^36^, ^40^. Although the two TE domains only shared 21.0% identity, the overall structures of IdgS‐TE and PadE‐TE, which were predicted using AlphaFold 2^41^, were very similar, and the conserved Ser residues for substrate binding in those two TE domains were well aligned in the same location (Figure S7A). However, further molecular docking analysis with the SNAC mimics of their corresponding substrates showed that the binding pockets of the two TE domains are clearly in different sizes and the proposed catalytic residues in PadE‐TE are not conserved in IdgS‐TE, indicating they may have different catalytic processes (Figure S7B,C).

### Preparation of (*S*)‐2 by immobilized IdgS‐Ox* R539A

For a better applicability, the reaction conditions of IdgS‐Ox* R539A were optimized. At the optimal condition (30°C, pH 9.0), IdgS‐Ox* R539A could produce (*S*)‐**2** at a yield of 20.8 ± 1.4%. We noticed that the enzyme was not very stable and precipitated at the late stage of the conversion. To improve the catalytic efficiency, we tried to stabilize the catalyst by enzyme immobilization. While the attempt to immobilize IdgS‐Ox* R539A to chitosan failed, IdgS‐Ox* R539A could be successfully immobilized to agarose and alginate with recovery rates of 55.5 ± 14.5% and 13.2 ± 5.1%, respectively. When 2.5% agarose was used as the carrier, the yield of (*S*)‐**2** could reach 32.3 ± 6.4% (*ee* >99%). The immobilized IdgS‐Ox* R539A retained about 50% catalytic activity after 1 h bioconversion process.

Although IdgS‐Ox* R539A exhibited excellent stereo‐specificity and generated enantiomerically pure (*S*)‐**2** as desired, its further applications were limited by the enzyme's instability. Recently, it was reported that **3** could be produced at titers approaching 90 g/l in a heterologous host, indicating that engineered cells have great potential in synthesizing the intermediate of **3**, (*S*)‐**2** ^42^. Therefore, the development of whole‐cell bioconversion systems and construction of (*S*)‐**2** high‐producing cell factories may be feasible ways to fulfill the demand for large scale preparation of this compound.

### One‐pot chemoenzymatic synthesis of **1**

As a proof of concept, we combined the bioconversion and the chemical condensation reactions to achieve one‐pot chemoenzymatic synthesis of **1**. The enzymatic conversion of L‐Gln to (*S*)‐**2** was first allowed to proceed with immobilized IdgS‐Ox* R539A under the optimal condition. The reaction mixture was then lyophilized and refluxed with phthalic anhydride, triethanolamine, and glacial acetic acid, which resulted in a 20.21 ± 3.5% yield of **1** (Figures 4D and S1D)^16^. Although it was just a preliminary trial, the successful production of **1** by this two‐step one‐pot synthesis paves a way for the preparation of this family of traditional chemical drugs by integrating the power of biological approaches.

Notably, a recent study of the Ox domain of BpsA (an IdgS isoenzyme from *Streptomyces lavendulae* ATCC 11924) showed that the BpsA TE‐null mutant (S1102A) could convert PCP‐tethered L‐Gln to a 5‐amino‐2,5‐dioxopentyl product, which implied that the PCP‐tethered L‐Gln could be oxidized by BspA‐Ox to 2,3‐dehydroglutaminyl^43^. The results indicated that, in addition to the off‐line oxidation activity, the Ox domain of indigoidine synthetase may also have online oxidation activity. Actually, due to the promiscuities of biosynthetic enzymes, many natural products can be made via multiple pathways^44^, ^45^, ^46^. Unfortunately, no evidence showed that the BpsA‐TE domain can convert PCP‐tethered dehydroglutaminyl into **3**, leaving whether **3** can be biosynthesized via route B an open question.

In summary, we showed that **3** can be biosynthesized via route A by dissecting the domain functions of IdgS and characterized (*S*)‐**2** as a biosynthetic intermediate, which allowed us to develop IdgS‐Ox* R539A as a biocatalyst for synthesizing (*S*)‐**2**, the pharmacophore of a number of drugs. As aforementioned, due to the interconversion between the **2** enantiomers, two major obstacles for applying compounds containing **2** as chiral drugs are the preparation of enantiomerically pure (*S*)‐**2** and stabilization of the (*S*)‐configuration in the following drug synthesis and administration processes. Hopefully, the IdgS‐based bioconversion system for (*S*)‐**2** preparation can help to overcome the first obstacle and facilitate the research on the second one.

## MATERIALS AND METHODS

### Strains, plasmids, and chemicals

Bacterial strains and plasmids used in this study are listed in Table S1. Racemic **2** was purchased from Fluorochem Ltd., UK. Standard **1** was purchased from Sigma‐Aldrich Co.

### DNA manipulation and sequence analysis

General DNA manipulations were performed as described^47^. PCR was performed with PrimeSTAR DNA polymerase (Takara) or *Taq* DNA polymerase (TransGene) according to the manufacturer's instructions. The primers used in this study (Table S2) were synthesized by Generay. DNA gel extraction and plasmid preparation kits were purchased from Omega Bio‐Tek (Norcross). Common DNA sequencing was performed by Biosune. The gene functional annotations were performed with BLAST (http://www.ncbi.nlm.nih.gov/blast). Multiple alignments were performed with CLUSTALW (https://www.ebi.ac.uk/Tools/msa/clustalw2/).

### Construction of plasmids for producing different recombinant proteins

The 3.9‐kb gene *idgS* was amplified using plasmid pCIM2002^27^ as the template with primer pair Idg‐F and Idg‐R and cloned into the *Nde*I/*Bam*HI sites of plasmid pET28a(+) to afford pET28a/*idgS*. The 4.6‐kb fragment containing genes *idgS* and *sfp* was amplified using plasmid pCIM2002 as the template with primer pair Idg‐F and Sfp‐R and cloned into the *Nde*I/*Hin*dIII sites of plasmid pET28a(+) to generate pET28a/*idgS‐sfp* for producing IdgS protein in its *holo*‐form.

The 0.8‐kb truncated fragment containing TE domain coding region and the 1.0‐kb truncated fragment containing T‐TE didomain coding region were amplified using plasmid pJRI02 (with codon‐optimized *idgS* gene) as the template with primer pairs TE‐F/TE‐R and T‐TE‐F/T‐TE‐R, respectively. Then, the PCR products were cloned into the *Nde*I/*Hin*dIII sites of pET28a(+) to generate the plasmids pET28a/*te* and pET28a/*t‐te* for producing the TE domain and T‐TE didomain, respectively.

To construct pET28a/idgS‐TE*‐sfp for producing mutant protein IdgS‐TE* S1102A, two fragments (3.3 and 0.55 kb) were first amplified using genomic DNA of *Streptomyces lavendulae* CGMCC 4.1386 as the template with primer pairs TEm‐U‐F/TEm‐U‐R and TEm‐D‐F/TEm‐D‐R, respectively. Next, phusion PCR was carried out with primer pair TEm‐U‐F/TEm‐D‐R using the above two fragments as templates, and then the PCR products were ligated into the *Nde*I/*Bam*HI sites of pET28a/*idgS‐sfp* by LIC strategy to afford pET28a/idgS‐TE*‐sfp ^48^.

To construct pET28a/idgS‐OxR*‐sfp for producing mutant protein IdgS‐Ox* R539A, two fragments (2 and 0.7 kb) were amplified using the genomic DNA as the template with primer pairs IdgS‐armF/ArgM‐armR and ArgM‐armF/IdgS‐armR, respectively, and then the two fragments were cloned into *Eco*RI/*Sal*I digested pET28a/*idgS‐sfp* to generate the desired plasmid pET28a/idgSoxR*‐sfp. The plasmid pET28a/*idgS‐OxS* ^*^ *‐sfp* (IdgS‐armF/SerM‐armR and SerM‐armF/IdgS‐armR) expressing the mutant gene idgS‐Ox* (S603A) was obtained using the same procedure to that of pET28a/*idgS‐OxR* ^*^ *‐sfp*.

### Protein expression and purification

The aforementioned plasmids were transformed into *E. coli* BL21 (DE3) to generate the corresponding recombinant strains for protein production. The recombinant proteins were overproduced and purified using the same procedure as follows. The recombinant strain was cultivated in LB medium at 37°C overnight to obtain seed culture. Then, 1.5 ml seed culture was inoculated into 150 ml LB, and then was allowed to grow at 37°C, 200 rpm until the OD_600_ reached 0.6–0.8. The cell culture was induced by 0.1–0.2 mM isopropyl‐*β*‐D‐thiogalactopyranoside and cultivated for another 18 h at 16°C, 180 rpm. After centrifugation, the cell pellets were resuspended in 10 ml lysis buffer (20 mM Tris‐HCl, 500 mM NaCl, 5 mM imidozale, 5% glycerol, pH 7.9) and lysed in an ice bath by ultrasonication (5 s pulse, 9.9 s break, 20 min in total). The cell debris was removed by centrifugation at 10,000*g* for 50 min, and the supernatant was then subjected to Ni‐NTA resin (Qiagen) to purify the His_6_‐tagged protein by affinity chromatography. The column was first washed with 10 ml lysis buffer followed by 10 ml wash buffer (20 mM Tris‐HCl, 500 mM NaCl, 60 mM imidazole, pH 7.9), and then the bound His_6_‐tagged proteins were eluted with elution buffer (20 mM Tris‐HCl, 500 mM NaCl, 250 mM imidazole, pH 7.9). The target protein was exchanged to 50 mM Tris‐HCl buffer (pH 9.0) with a PD‐10 column (GE Healthcare). Finally, the target protein was concentrated by 10 kDa size‐exclusion filters (Amicon) and stored in storage buffer (50 mM Tris‐HCl, pH 9.0, 20% glycerol) at −80°C. The protein concentration was measured by the Bradford method^49^.

### Spectrometric analysis of IdgS and IdgS‐TE* S1102A under anaerobic condition

To quickly assess the route by which indigoidine is biosynthesized, the abilities of IdgS and IdgS‐TE* S1102A to reduce FMN to FMNH_2_ were monitored under anaerobic conditions. The reactions were carried out in an oxygen‐free anaerobic box to prevent the reduced FMNH_2_ from re‐oxidation. The 200 μl reaction mixture consisted of 0.1 mM IdgS or IdgS‐TE* S1102A, 10 mM ATP, 10 mM MgCl_2_, 10 mM L‐Gln, and 50 mM Tris‐HCl buffer (pH 8.5). The UV–vis spectrum of each reaction was recorded after 3 h. The negative control was the reaction without L‐Gln. Spectrophotometric determinations were performed in the quartz cuvette (1 cm pathlength, and 0.5 ml volume). The absorbance difference was determined using UV–vis spectroscopy by monitoring the absorption at 350 and 450 nm, which changes depending on the redox state of the cofactor FMN.

#### *In vitro* assay of IdgS and IdgS‐TE* S1102A

A typical 100 μl reaction consisting of 5 μM IdgS or IdgS‐TE* S1102A (Apo‐ IdgS‐TE* S1102A), 10 mM ATP (ADP or AMP), 10 mM MgCl_2_, 10 mM L‐Gln or **2**, and 50 mM Tris‐HCl (pH 8.5) was carried out at 30°C for 1 h. The reaction mixture was quenched by adding an equal volume of CHCl_3_. After centrifugation, the middle protein layer was collected and eluted with dimethyl sulfoxide (DMSO) to dissolve the adherent indigoidine. The supernatant was collected for analysis of ATP, ADP, or AMP consumption.

#### *In vitro* assay of IdgS‐Ox* R539A

A typical 100 μl reaction consisting of 5 μM IdgS‐Ox* R539A, 10 mM ATP, 10 mM MgCl_2_, 2 mM L‐Gln, and 50 mM Tris‐HCl (pH 9.0) was carried out at 30°C for 1 h. The reaction mixture was quenched by adding an equal volume of CHCl_3_. After centrifugation, the supernatants were collected and then subjected to precolumn derivatization with Fmoc‐Cl for HPLC analysis^50^.

### Spectroscopic analysis

HPLC analyses were carried out with an Apollo C18 column (5 μm, 4.6 mm × 250 mm; Alltech) on a Shimadzu HPLC system (Shimadzu). For analysis of **3**, the column was developed with acetonitrile and water containing 0.1% trifluoroacetic acid at a flow rate of 1.0 ml/min. The percentage of acetonitrile was changed linearly from 10% to 90% for 40 min, from 90% to 100% for 5 min, 100% for 5 min, and from 100% to 10% for 10 min. The detection wavelength was 600 nm. For analysis of **2**‐Fmoc, the column was developed with acetonitrile and water containing 0.1% formic acid at a flow rate of 1.0 ml/min. The percentage of acetonitrile was changed linearly from 40% to 50% for 40 min, from 50% to 100% for 5 min, 100% for 5 min, and from 100% to 40% for 10 min. The detection wavelength was 210 nm. For analysis of **2**‐DAA, the column was developed with acetonitrile and water containing 0.1% trifluoroacetic acid at a flow rate of 1.0 ml/min. The percentage of acetonitrile was 23% for 50 min. The detection wavelength was 340 nm. For analysis of **1**, the column was developed with acetonitrile and water containing 0.1% trifluoroacetic acid at a flow rate of 1.0 ml/min. The percentage of acetonitrile was changed linearly from 20% to 40% for 40 min, from 40% to 100% for 5 min, 100% for 5 min, and from 100% to 20% for 10 min. The detection wavelength was 285 nm.

Analysis of the ATP consumption in the reaction of IdgS‐TE* S1102A using **2** as a substrate was performed with a Dionex CarboPac^TM^ PA1 BioLC^TM^ column (4 mm × 250 mm; Thermo Fisher Scientific). The column was developed with water and solvent A (water containing 1 M ammonium acetate) at a flow rate of 1.0 ml/min. The percentage of solvent A was changed linearly from 25% to 45% for 5 min, from 45% to 65% for 13 min, from 65% to 90% for 2 min, 90% for 11 min, and from 90% to 25% for 2 min. The detection wavelength was 254 nm.

Liquid chromatography‐mass spectrometry (LC‐MS) was performed on an Agilent 1260/6460 Triple‐Quadrupole LC/MS system with the electrospray ionization source. HPLC‐high resolution mass spectrometry (HPLC‐HR‐MS) was performed on an Agilent 1260 HPLC/6520 QTOF‐MS instruments with the electrospray ionization source. Nuclear magnetic resonance spectroscopy (NMR) spectra were recorded at room temperature on a Bruker Advance 500M NMR spectrometer.

### One‐pot chemoenzymatic synthesis of **1**

The scaled‐up 10 ml reaction consisting of immobilized IdgS‐Ox* R539A, 10 mM ATP, 10 mM MgCl_2_, 10 mM L‐Gln, and 50 mM Tris‐HCl (pH 9.0) was carried out at 30°C for 2 h. After centrifugation and filtration, the supernatants were collected and lyophilized for the chemical synthesis of **1**. The lyophilized powder containing (*S*)‐**2** from enzymatic reactions was added to a mixture of phthalic anhydride (7.5 mg, 0.05 mM), Et_3_N (0.018 ml, 0.13 mM), and glacial acetic acid (0.6 ml, 0.01 mM)^16^. The mixture was stirred under reflux conditions for 2 h, and then dissolved in an equal volume of DMSO. Production of **1** was quantified by HPLC analysis. HR‐MS (*m*/*z*): calculated for C_13_H_10_N_2_O_4_ [M + H]^+^ 259.0713; found: 259.0706.

## AUTHOR CONTRIBUTIONS

Zhilong Zhang performed the *in vitro* assays and completed the one‐pot‐pot chemoenzymatic synthesis of thalidomide. Pengwei Li constructed IdgS‐based proteins and analyzed the data. Min Wang synthesized the substrate mimics. Yong Tao, Yan Zhang, and Bian Wu provided experimental materials and gave constructive suggestions for this study. Pengwei Li, Guohui Pan, and Yihua Chen wrote the manuscript. Yihua Chen conceived the project and supervised this study.

## ETHICS STATEMENT

Not applicable.

## CONFLICT OF INTERESTS

The authors declare no conflict of interests.

## Supporting information

Supplementary information.

## Data Availability

All data are contained within the article.
