# Supplementary material for: (S)‐3‐aminopiperidine‐2,6‐dione is a biosynthetic intermediate of microbial blue pigment indigoidine
Source: mLife. 2022 Jun 21;1(2):146–55. doi: 10.1002/mlf2.12023 (PMC10989907; doi:10.1002/mlf2.12023)
Supplement: Supplementary file 1 — Supplementary information. [file MLF2-1-146-s001.docx]

**Supporting Information**

**(*S*)-3-aminopiperidine-2,6-dione is a biosynthetic intermediate of microbial blue pigment indigoidine**

Table of Contents

| **Methods ……………………………………………………………………………………………....................** | 2 |
| --- | --- |
| **Table S1.** Bacterial strains and plasmids **………………………………………………………………………..** | 5 |
| **Table S2**. Primers used in this study **……………………………………………………………………………..** | 6 |
| **Figure S1.** HR-MS analyses of indigoidine (A), **2**-Fmoc (B), **2**-DAA (C), and thalidomide (D) **……………...** | 7 |
| **Figure S2.** SDS-PAGE analysis of IdgS and the related recombinant proteins and sequence analysis of the Ox domain of IdgS**…………………………………………………………………………………………………...** | 8 |
| **Figure S3.** HPLC analysis and comparison of ATP changes in IdgS and IdgS-TE^*^ S1102A catalyzed reactions | 9 |
| **Figure S4.** HPLC analysis and comparison of **3** in IdgS-TE^*^ S1102A (A) and apo-IdgS-TE^*^ S1102A (B)-catalyzed reactions**.…………………………………………………..…………………………………………..** | 10 |
| **Figure S5.** HR-MS and NMR spectra of L-Gln-SNAC**.………………………………………………………….** | 11 |
| **Figure S6.** Reaction conditions optimization and apparent kinetic analysis of IdgS-Ox^*^ R539A**………………** | 12 |
| **Figure S7.** Structural comparison of IdgS-TE and PadE-TE**.…………………………………………………….** | 13 |
| **References ……………………………………………………………………………………………………....** | 14 |

**In vitro assay of the truncated IdgS T-TE didomain protein**

A typical 100 *μ*L reaction consisting of 5 *μ*M IdgS T-TE (apo- or holo- form), 0.2 mM L-Gln-*S*-N-acetyl-cysteamine (L-Gln-SNAC) as a mimic substrate, and 50 mM Tris-HCl (pH 8.5), was carried out at 30 °C for 2 h, and quenched by adding equal volume of CHCl_3_. After centrifugation, the supernatants were collected and subjected to pre-column derivatization with Fmoc-Cl for HPLC analysis (1). A typical derivatization reaction that consists of 10 *μ*L supernatant of the enzymatic reaction, 10 *μ*L Na_2_CO_3_ (40 mM), and 30 *μ*L Fmoc-Cl (40 mM) was performed at room temperature for 1 h. The mixture was quenched and extracted by ethyl acetate, and then dissolved in methanol for HPLC and MS analysis.

**FDAA derivatization of 2**

The 170 *μ*L reaction mixture consisting of 30 mM **2** (stock in 0.1 M HCl), 0.47 M NaHCO_3_, and 26 mM FDAA (stock in acetone), was allowed to proceed at 50 °C for 1 h. After cooled to room temperature, the reaction mixture was quenched by adding 40 *μ*L HCl (1 M) (2). The reaction mixture was dried in vacuum, and the products were dissolved in 40 *μ*L acetonitrile for HPLC and MS analysis.

**Enzyme immobilization of** **IdgS-Ox^*^ R539A**

Immobilization of IdgS-Ox^*^ R539A on different supports was carried out to improve the enzyme stability. The IdgS-Ox^*^ R539A mutant protein was tested for immobilization on four different supports (agarose, low melting-point agarose, alginate and chitosan) [3-6]. For agarose as the carrier, 10 *μ*L IdgS-Ox^*^ R539A mutant protein (10 mM) was mixed with 10 *μ*L agarose solution (2.5% w/v, cooled to room temperature). After solidification for 1 h at 4 °C, the gel was cut into small beads and washed twice with 50 mM Tris-HCl buffer (pH 9.0) containing 100 mM NaCl and 10% glycerol, and then stored in Tris-HCl buffer (pH 9.0, 4 °C) before use (3).

The procedure of enzyme immobilization with low melting-point agarose (agarose, 5% w/v) was the same as agarose. For the alginate carrier, alginate solid was added into 10 *μ*L of 10 mM IdgS-Ox^*^ R539A to a final concentration of 5% (w/v), stirred while adding and kept at 40-50 °C until it was dissolved. Then, the colloidal solution was dropwise added into 5 mL of 3% CaCl_2_. After solidification, the immobilized IdgS-Ox^*^ R539A protein was washed extensively with double distilled water to remove residual CaCl_2_ (4).

For enzyme immobilization, chitosan microparticles were prepared first. Chitosan (2 g) was dissolved in acetic acid solution (30 ml, 1% w/v) and dropwise added into NaOH (1 M) to form microparticles. After washed extensively with Tris-HCl buffer (pH 9.0), 5 mg chitosan microparticles was added to glutaraldehyde (10 ml, 1% w/v) to activate the microparticles for about 2 h. The microparticles were then washed twice with the same Tris-HCl buffer and added to 10 *μ*L IdgS-Ox^*^ R539A protein (10 mM). After standing for 12 h at 4 °C, the IdgS-Ox^*^ R539A microparticles were washed and stored at 4 °C for enzyme reaction (5). The recovery activities were calculated by detecting the activities of the immobilized enzymes and the activity of the initial enzyme solution (6).

**Synthesis of L-Gln-S-N-acetyl-cysteamine (L-Gln-SNAC)**

**SNAC preparation**

Cysteamine hydrochloride (3.5 g, 30 mM), potassium hydroxide (1.5 g, 30 mM) and sodium bicarbonate (6.75 g, 80 mM) were added to 50 mL water. Acetic anhydride (2.28 mL) was then added dropwise into the reaction mixture over 5 min. The reaction was allowed to stir at room temperature for 15 min, and then was quenched with 1 M HCl. The product was extracted with ethyl acetate, dried with Na_2_SO_4_, filtered, and concentrated *in vacuum* to yield a clear liquid (3.0 g). The crude SNAC product was used for following synthesis without purification.

**Boc-L-Gln-SNAC preparation**

A mixture of DCC (0.13 g, 0.63 mM) and HOBt (0.17 g, 1.26 mM) in THF was added to Boc-L-Gln (0.21 g, 0.85 mM) in THF, and then SNAC (0.1 g, 0.84 mM) was added into the mixture. After the reaction mixture was stirred for 1 h at room temperature, potassium carbonate (0.06 g, 0.44 mM) was added. After the reaction was stirred for another 3 h at room temperature, it was extracted with ethyl acetate, dried with Na_2_SO_4_, filtered, and concentrated *in vacuum*. The crude product was purified by flash chromatography to afford Boc-L-Gln-SNAC (0.2 g, 67%).

**L-Gln-SNAC preparation**

The Boc group was removed by using TFA/DCM (1:1). After incubation for 1 h at room temperature, the reaction was taken up in DCM and washed with NaHCO_3_. The organic layer was dried with Na_2_SO_4_, filtered, and concentrated *in vacuum*. The crude product was purified by flash chromatography to afford L-Gln-SNAC (0.1 g, 71%). **^1^H NMR** (500 MHz, DMSO-*_d6_*) *δ* 4.11 (t, *J* =5.0Hz, 1H), 3.29 (m, 2H), 2.76 (m, 2H), 1.80 (s, 3H), 1.70 (m, 2H), 1.63 (m, 2H). **^13^C NMR** (125 MHz, DMSO-*_d6_*): *δ* 197.4, 171.1, 170.4, 52.0, 42.6, 30.8, 26.2, 23.8, 22.8. **HR-MS** (*m*/*z*): calculated for C_9_H_17_N_3_O_3_S [M+H]^+^ 248.1063, found: 248.1071. **^1^H NMR** and **^13^C NMR** spectra, see **Figure S4.**

**Synthesis of (*S*)-2** (7)

**Cbz-(*S*)-2 preparation**

Compounds ((benzyloxy)carbonyl)-L-glutamine (0.1 g, 0.38 mM) and N-hydroxysuccinimide (0.04 g, 0.35 mM) were dissolved in 0.3 mL THF/DMF (5:1). The solution was cooled to -78 °C, and then DCC (0.08 g, 0.39 mM) was added in one portion. The reaction mixture was allowed to gradually warm to room temperature and stirred overnight. After removing the precipitate by filtration, the filtrate was concentrated *in vacuum*. The residue was taken up in CHCl_3_ (0.5 mL) and heated to reflux for 3 h. After being cooled to room temperature, volatiles were removed *in vacuum*, and the mixture was extracted with DCM. The organic phase was dried over sodium sulfate (anhydrous), filtered, and concentrated *in vacuum*. The residue was purified by silica gel column chromatography to afford Cbz-(*S*)-**2** (0.04 g, 40.4%).

**(*S*)-2 preparation**

Palladium-carbon (2 mg, 10% on carbon) was added to a solution of Cbz-(*S*)-**2** (0.04 g, 0.15 mM) in methanol (1 mL). The reaction vessel was sealed and changed to hydrogen atmosphere, and stirred at room temperature for 4 h. After removing palladium-carbon by filtration, the filtrate was concentrated *in vacuum* to give (*S*)-**2** (0.01 g, 52.6%) as a white solid: [α] D^25^-51.99 (c 0.03, MeOH). (*S*)-**2**-DAA, **HR-MS** (*m*/*z*): calculated for C_14_H_16_N_6_O_7_ [M+H]^+^ 381.1153, found: 381.1157.

**Table S1.** **Bacterial strains and plasmids**

| **Strains or plasmids** | **Characteristics** | **Reference or source** |
| --- | --- | --- |
| ***Escherichia coli*** |  |  |
| DH5α | General cloning host | Invitrogen |
| BL21 (DE3) | Host for protein expression | Novagen |
| ***Streptomyces*** |  |  |
| *S. lavendulae* CGMCC 4.1386  **Plasmids** | Indigoidine producing wild-type strain | (8) |
| pCIM2002 | Apr^r^, used for amplification of fragment containing *idgS* and *sfp* | (9) |
| pET28a | Kan^r^, protein production vector | Novagen |
| pJRI02 | Cm^r^, used for amplification of fragments from *idgS* | (10) |
| pET28a/*idgS*-*sfp* | Kan^r^, for producing *N*-His_6_-tagged IdgS in its holo-form | This work |
| pET28a/*te* | Kan^r^, for producing *N*-His_6_-tagged TE domain | This work |
| pET28a/*t-te* | Kan^r^, for producing *N*-His_6_-tagged T-TE domains | This work |
| pTGE33 | Kan^r^, for producing *N*-His_6_-tagged Sfp | (11) |
| pET28a/*idgS-TE^*^-sfp* | Kan^r^, for producing *N*-His_6_-tagged IdgS-TE^*^ S1102A | This work |
| pET28a/*idgS-OxR^*^-sfp* | Kan^r^, for producing *N*-His_6_-tagged IdgS-Ox^*^ R539A | This work |
| pET28a/*idgS-OxS^*^-sfp* | Kan^r^, for expressing the mutant gene *idgS-Ox*^*^ (S603A) | This work |

Kan^r^, kanamycin resistance; Cm^r^, chloramphenicol resistance.

**Table S2. Primers used in this study**

| **Primers** | **Sequences (5′ to 3′)** | **Application** |
| --- | --- | --- |
| Idg-F | actcgacatatgactcttcaggagaccagcgtgc | Amplification of the gene *idgS* (*Nde*I and *Bam*HI)  Amplification of the gene cassette containing *idgS* and *sfp* with Idg-F (*Nde*I and *Hin*dIII) |
| Idg-R | tgactaggatccctactctccgagcaggtagcggatg |  |
| Sfp-R | ggccgcaagcttttataaaagctcttcgtacg |  |
| TE-F | atcagtcatatgcgtccggttctgtgttgg | Amplification of the thioesterase domain region of IdgS (*Nde*I and *Hin*dIII) |
| TE-R | acgtgcaagctttcacagcagatagcgaatatgtttaacc |  |
| T-TE-F | atcagtcatatgctggttgaacgcccgtttgt | Amplification of the T-TE didomain coding region of IdgS (*Nde*I and *Hin*dIII) |
| T-TE-R | acgtgcaagctttcattcacccagcagatagcgaat |  |
| TEm-U-F | tgccgcgcggcagccatatgactcttcaggagaccagcg | Amplification of the partial fragment (N-terminal) of *idgS-TE^*^* |
| TEm-U-R | aaggcgacgcgggcgccgaaggcgtagccccacagggtgtag |  |
| TEm-D-F | ctacaccctgtggggctacgccttcggcgcccgcgtcgcctt | Amplification of the partial fragment (C-terminal) of *idgS-TE^*^* |
| TEm-D-R | agatctgacagagtggatccctactctccgagcaggtagcggatg |  |
| IdgS-armF | tcaactcctcctcgtacgcggtcgaccccgccgagctgg | Amplification of the upstream and downstream fragments of gene *idgS* |
| IdgS-armR | gctcacggaacgagtactcgaattcgtaggtctgccccac |  |
| ArgM-armF | gcgcagccgggtcttcgccgcaaagacgtaccgcttctac | Amplification of the upstream and downstream fragments of the mutated gene *idgS-oxR^*^* |
| ArgM-armR | gtagaagcggtacgtctttgcggcgaagacccggctgcgc |  |
| SerM-armF | ctgcccaagtacggctacgccgcaccgggcgcgctgtacgcgacg | Amplification of the upstream and downstream fragments of the mutated gene *idgS-oxS^*^* |
| SerM-armR | cgtcgcgtacagcgcgcccggtgcggcgtagccgtacttgggcag |  |

**Figure S1. HR-MS analyses of indigoidine (A), 2-Fmoc (B), 2-DAA (C) and thalidomide (D).** indigoidine (**3**), **HR-MS** (*m*/*z*): calculated for C_10_H_8_N_4_O_4_ [M+H]^+^ 249.0618, found: 249.0614; **2**-Fmoc, **HR-MS** (*m*/*z*): calculated for C_20_H_18_N_2_O_4_ [M+H]^+^ 321.1339, found: 321.1345; **2**-DAA, **HR-MS** (*m*/*z*): calculated for C_14_H_16_N_6_O_7_ [M+H]^+^ 381.1153, found: 381.1157; thalidomide (**1**), **HR-MS** (*m*/*z*): calculated for C_13_H_10_N_2_O_4_ [M+H]^+^ 259.0713, found: 259.0706.


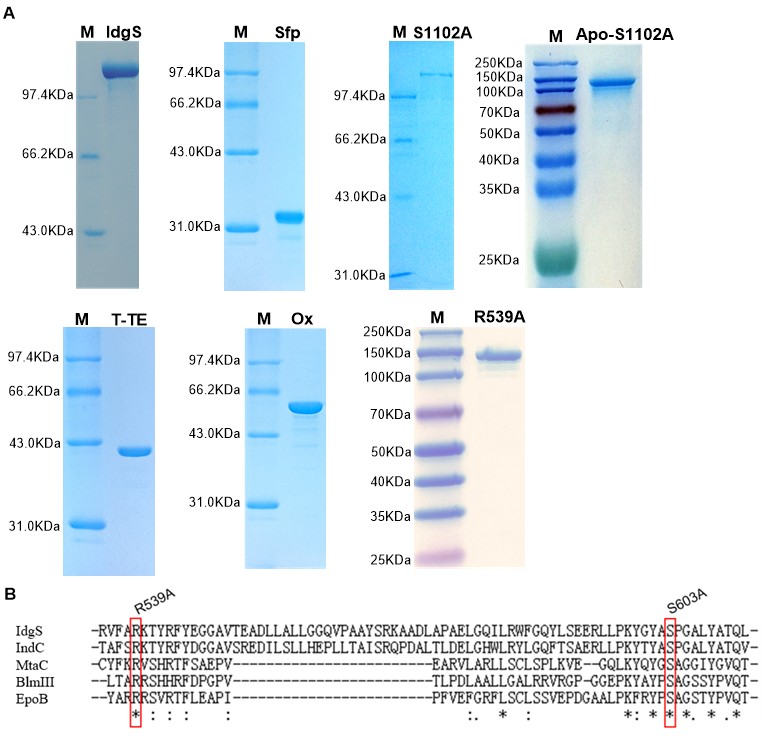


**Figure S2.** **SDS-PAGE analysis of IdgS and the related recombinant proteins and sequence analysis of the Ox domain of IdgS.** (A) SDS-PAGE analysis of IdgS, Sfp, IdgS-TE^*^ S1102A, Apo- IdgS-TE^*^ S1102A, the truncated IdgS T-TE didomain protein, the truncated IdgS Ox didomain protein and IdgS-Ox^*^ R539A. (B) Sequence alignment of the Ox domain of IdgS and its homologous domains from other NPRSs (mutated sites are highlighted in red rectangles). IdgS, indigoidine synthetase from *Streptomyces lavendulae* CGMCC 4.1386 (WP_030237949); IndC, indigoidine synthetase from *Dickeya chrysanthemi* sp. 3937(CAB87990); MtaC, NRPS enzyme from *Stigmatella aurantiaca* DW4/3-1(AAF19811); BlmIII, bleomycin peptide synthetase NRPS from *Streptomyces verticillus* ATCC15003 (AAG02365); EpoB, epothilone synthetase from *Sprangium cellulosum* (ABB92691).

**Figure S3. HPLC analysis and comparison of ATP changes in IdgS and IdgS-TE^*^ S1102A catalyzed reactions.**  ATP was not consumed by IdgS-TE^*^ S1102A whether the substrate **2** was added or not. Similarly, ATP was not consumed in the assay containing boiled IdgS-TE^*^ S1102A.


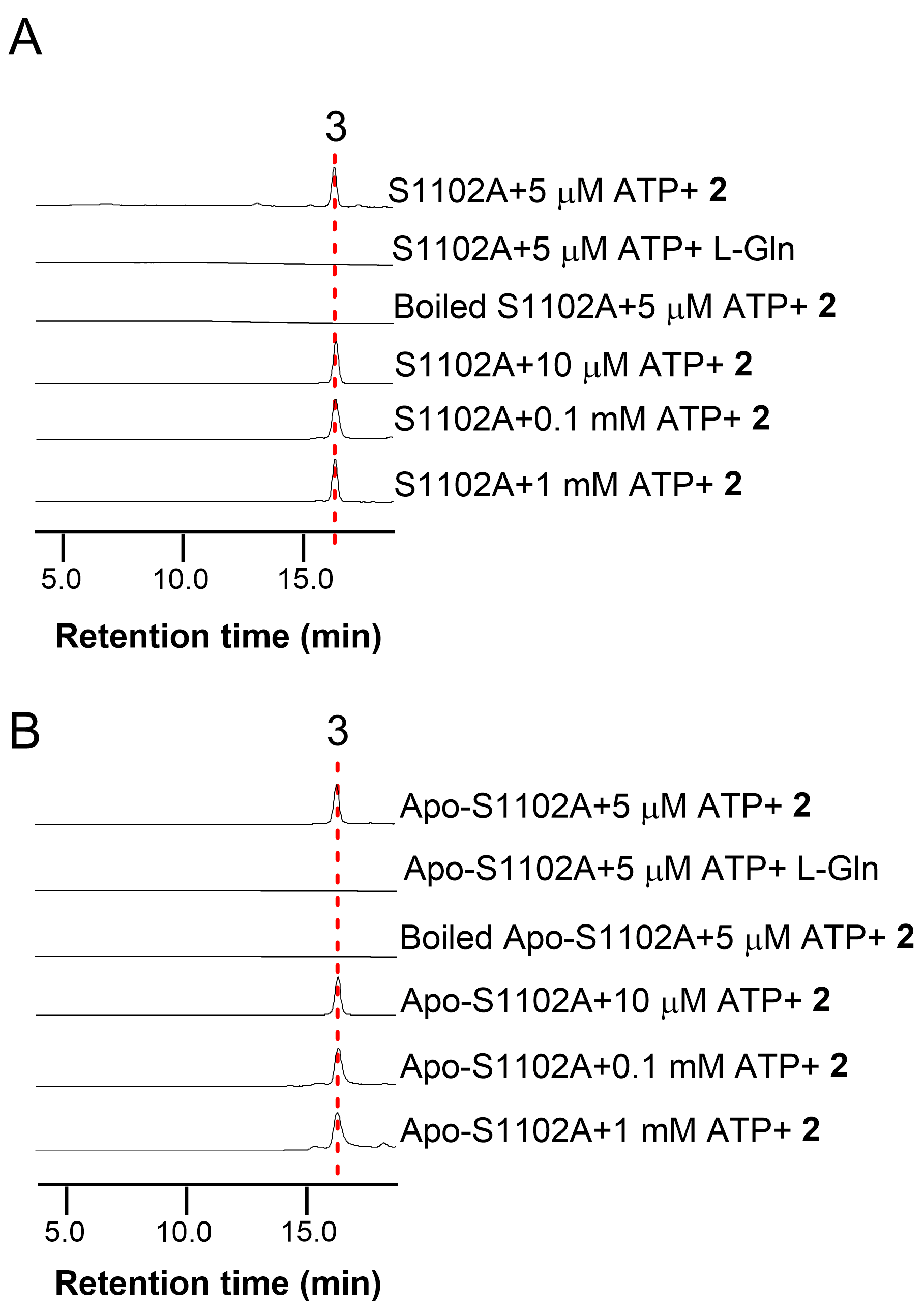


**Figure S4. HPLC analysis and comparison of 3 in** **IdgS-TE^*^ S1102A (A) and Apo-IdgS-TE^*^ S1102A (B)-catalyzed reactions.** Each reaction mixture (100 *μ*L) containing 5 *μ*M IdgS- TE^*^ S1102A or Apo- IdgS- TE^*^ S1102A, 10 mM **2** or L-Gln, 10 mM MgCl_2_, 100 mM NaCl, and 50 mM Tris-HCl buffer (pH 8.5) was performed for 2 h at 30 °C. The production of **3** was not dependent on concentrations of ATP.

**Figure S5. HR-MS and NMR spectra of L-Gln-SNAC.** (A) Structure, (B) HR-MS spectrum, (C) ^1^H-NMR spectrum, and (D) ^13^C NMR spectrum of L-Gln-SNAC.

**
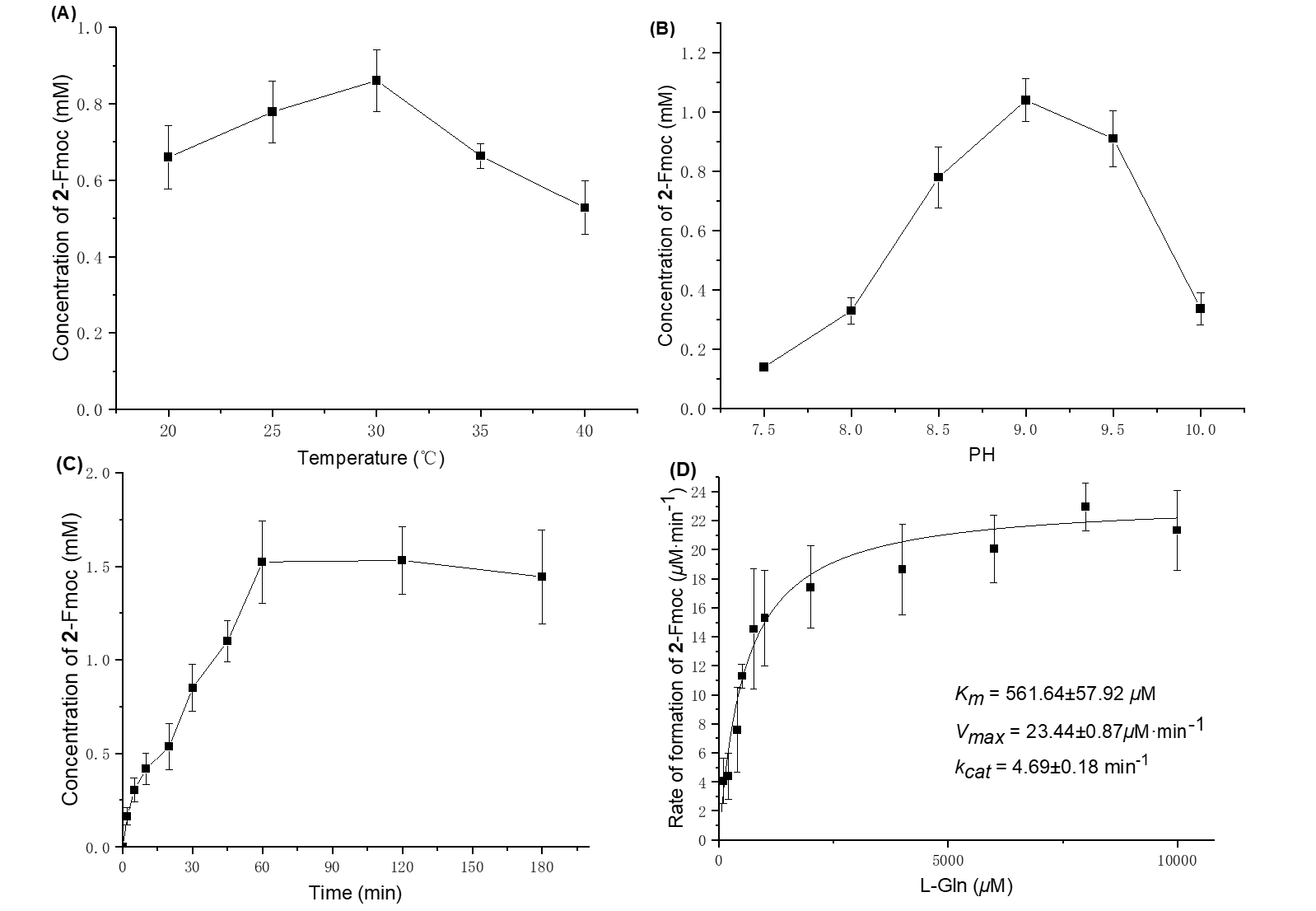
**

**Figure S6.** **Reaction condition optimization and apparent kinetic analysis of IdgS-Ox* R539A.** (A) Optimization of the temperature for IdgS-Ox* R539A catalyzed reactions. IdgS-Ox* R539A assays were performed for 0.5 h in a reaction mixture (100 *μ*L) containing 5 *μ*M IdgS-Ox* R539A, 10 mM L-Gln, 10 mM ATP, 10 mM MgCl_2_, 100 mM NaCl, and 50 mM Tris-HCl buffer (pH 9.0) with temperatures ranging from 20-40 °C. (B) Optimization of pH for the IdgS-Ox* R539A catalyzed reaction. Each reaction was carried out at 30 °C for 0.5 h in Tris-HCl buffers ranging from pH 7.5 to 10.0; (C)Time-course of IdgS-Ox* R539A catalyzed reaction; (D) Apparent kinetic analysis of IdgS-Ox* R539A. L-Gln was set as a variable substrate in concentrations of 0, 0.05, 0.1, 0.2, 0.4, 0.5, 0.75, 1, 2, 4, 6, 8, 10 mM. Enzymatic assays were performed in 50 mM Tris-HCl buffer (pH 9.0) containing 10 mM ATP, 10 mM MgCl_2_, 100 mM NaCl, and 5 *μ*M IdgS-Ox* R539A at 30 °C for 0.5 h in triplicate. The kinetic parameters were calculated by nonlinear regression analysis of substrate concentration versus initial velocity plots using Origin 8.0 software.

*
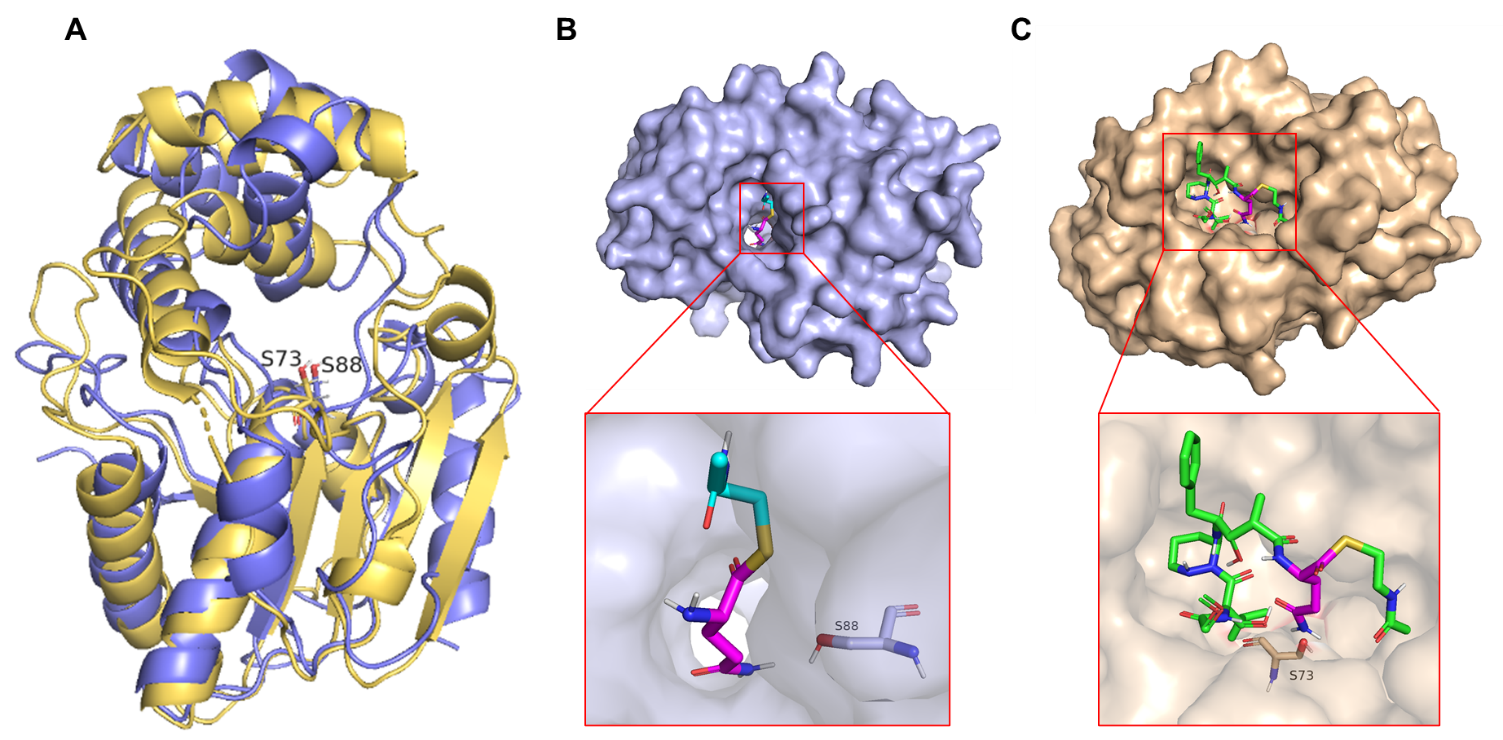
*

**Figure S7. Structural comparison of IdgS-TE and PadE-TE.** (A) Superposition of the overall structures of IdgS-TE (purple) and PadE-TE (yellow). The conserved Ser residues for substrate binding are labeled with letters (S73 from PadE-TE and S88 from IdgS-TE); (B) Molecular docking model of IdgS-TE with L-Gln-SNAC. The scaled-up inset shows the binding pocket (L-Gln-SNAC is shown as sticks and L-Gln moiety is indicated in magenta); (C) Molecular docking model of PadE-TE with padanamide-SNAC. The scaled-up inset shows the binding pocket (Padanamide-SNAC is shown as sticks and Gln moiety is indicated in magenta).

**References**

1. Tromp RA, Hoeven M, Amore A, et al. Synthesis of Fmoc-protected (2S,3S)-2-hydroxy-3-amino acids from a furyl substituted chiral cyanohydrin. Tetrahedron: Asymmetry 2003; 14(12): 1645-1652.
2. Bhushan R, Bruckner H. Marfey’s reagent for chiral amino acid analysis: a review. Amino Acids 2004; 27: 231-247.
3. Prakash O, Jaiswal N. Immobilization of a thermostable-amylase on agarose and agar matrices and its application in starch stain removal. World Appl Sci J 2011; 13(3): 572-577.
4. Ratanapongleka K, Punbut S. Removal of acetaminophen in water by laccase immobilized in barium alginate. Environ Technol 2018; 39(3): 336-345.
5. Dos Santos JCS, Bonazza HL, Matos LJBL, et al. Immobilization of CALB on activated chitosan: application to enzymatic synthesis in supercritical and near-critical carbon dioxide. Biotechnol Rep (Amst) 2017; 14: 16-26.
6. Sheldon RA, van Pelt S. Enzyme immobilisation in biocatalysis: why, what and how. Chem Soc Rev 2013; 42(15): 6223-6235.
7. Long B, Tang S, Chen L et al. Total synthesis of padanamides A and B. Chem Commun 2013; 49: 2977-2979.
8. Shirling ET, Gottlieb D. Method for characterization of *Streptomyces* species. Int J Syst Bacteriol 1966; 16: 313-340.
9. Li PW, Li J, Guo ZY et al. An efficient blue-white screening based gene inactivation system for *Streptomyces*. Appl Microbiol Biotechnol 2015; 99(4): 1923-1933.
10. Xie ZJ, Zhang Z, Cao ZJ et al. () An external substrate-free blue/white screening system in *Escherichia coli*. Appl Microbiol Biotechnol 2017; 101: 3811-3820.
11. Fang J, Zhang Y, Huang L et al. Cloning and characterization of the tetrocarcin a gene cluster from *Micromonospora chalcea* NRRL 11289 reveals a highly conserved strategy for tetronate biosynthesis in spirotetronate antibiotics. J Bacteriol 2008; 190: 6014-6025.
